# Segmentation and Tracking of Anticyclonic Eddies during a Submarine Volcanic Eruption Using Ocean Colour Imagery

**DOI:** 10.3390/s150408732

**Published:** 2015-04-14

**Authors:** Javier Marcello, Francisco Eugenio, Sheila Estrada-Allis, Pablo Sangrà

**Affiliations:** 1 Instituto de Oceanografía y Cambio Global, Universidad de Las Palmas de G.C., Campus Universitario de Tafira, 35017 Las Palmas de Gran Canaria, Spain; E-Mails: feugenio@dsc.ulpgc.es (F.E.); pablo.sangra@ulpgc.es (P.S.); 2 Departamento de Física, Universidad de Las Palmas de Gran Canaria, 35017 Las Palmas de Gran Canaria, Spain; E-Mail: sheila.estrada103@doctorandos.ulpgc.es

**Keywords:** volcano, eddies, segmentation, clustering, thresholding, watershed, MODIS, MERIS, Worldview-2

## Abstract

The eruptive phase of a submarine volcano located 2 km away from the southern coast of El Hierro Island started on October 2011. This extraordinary event provoked a dramatic perturbation of the water column. In order to understand and quantify the environmental impacts caused, a regular multidisciplinary monitoring was carried out using remote sensing sensors. In this context, we performed the systematic processing of every MODIS and MERIS and selected high resolution Worldview-2 imagery to provide information on the concentration of a number of biological, physical and chemical parameters. On the other hand, the eruption provided an exceptional source of tracer that allowed the study a variety of oceanographic structures. Specifically, the Canary Islands belong to a very active zone of long-lived eddies. Such structures are usually monitored using sea level anomaly fields. However these products have coarse spatial resolution and they are not suitable to perform submesoscale studies. Thanks to the volcanic tracer, detailed studies were undertaken with ocean colour imagery allowing, using the diffuse attenuation coefficient, to monitor the process of filamentation and axisymmetrization predicted by theoretical studies and numerical modelling. In our work, a novel 2-step segmentation methodology has been developed. The approach incorporates different segmentation algorithms and region growing techniques. In particular, the first step obtains an initial eddy segmentation using thresholding or clustering methods and, next, the fine detail is achieved by the iterative identification of the points to grow and the subsequent application of watershed or thresholding strategies. The methodology has demonstrated an excellent performance and robustness and it has proven to properly capture the eddy and its filaments.

## 1. Introduction

Mesoscalar oceanic eddies are ubiquitous structures that can be found anywhere in the world’s oceans, with a predominance of anticyclonic over cyclonic vortexes [1]. Several studies [2,3,4] point out that these mesoscalar structures can exert an important role on the global climate and local oceanic dynamic due to its influence in the transport of ocean heat and momentum, as well as, in the enhancement of vertical shear mixing and both biotic and abiotic tracers. 

In the North East Atlantic Ocean, the presence of the Canary Archipelago acts as an obstacle to the oceanic and atmospheric flows, originating a rich mesoscale variability mainly represented by anticyclonic and cyclonic eddies spun off from the islands’ flanks [5,6]. The mechanisms of such variability are invoked through the perturbation of the Canary Current and Trades Winds by the tall topography islands such as El Hierro [7]. Eddy radius varies between the origin island radius (~12–25 km) at their initial stage and c.a. 75 km at their dissipative stage [5]. They are between 300 and 700 m depth depending on their intensity [8]. Anticyclonic eddies are initially more intense with a rotation rate of about 3 days whereas the initial rotation rate for cyclones is ca. 5 days [6]. Canary Island eddies are long-lived eddies (>4 months) being at the origin of the Canary Eddy Corridor which is the main pathway for long-lived eddies in the subtropical North Atlantic Ocean [9], therefore they have not only a local impact but also a remote impact.

In this context, active and passive satellite sensors have been operating to investigate the variability of mesoscale features in the ocean. In particular, sea level anomaly fields from altimeters [10] have systematically been used to detect and monitor eddies. Some recent studies can be found in [11,12,13,14,15]. Unfortunately, these altimeter products have spatial resolution of tens of kilometres and, thus, they are suitable for global studies but are not adequate to perform detailed analysis at submesoscale level, to analyse their filaments or to study eddies with horizontal scales below 50 km, which is close to twofold the climatological first baroclinic Rossby radius of deformation for the Canary Islands region. This scale is the natural scale for mesoscale eddies at their initial stage and decreases with increasing latitude. However, these fine studies can be overcome from space by optical/infrared sensors [16,17,18,19]. In this way, satellite derived full resolution data of chlorophyll-a concentration or sea surface temperature can be used to thoroughly characterize eddies. Unfortunately, not all these structures can be clearly seen in ocean colour or thermal imagery, as this depends on which way they rotate, apart from the climatological inconvenience. Anti-cyclonic eddies in the northern hemisphere are particularly difficult to detect and analyse with these data.

**Figure 1:**
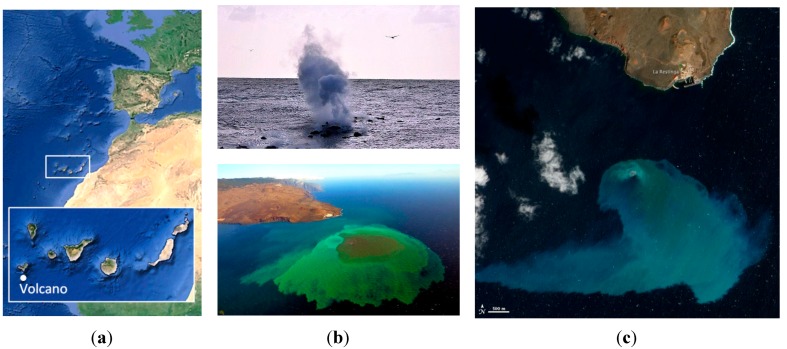
Submarine volcano eruption at El Hierro Island during October 2011: (**a**) location of the underwater volcano; (**b**) photographs during the eruption; and (**c**) satellite image by NASA EO-1 ALI^©^ sensor.

The eruption of a submarine volcano at El Hierro Island provided a unique and outstanding source of tracer that enabled us to study a variety of structures. El Hierro, an island off the Atlantic Ocean coast of North Africa was rocked by thousands of tremors and earthquakes since July 2011. On October 10 of the same year an underwater volcanic eruption started 300 meters below sea level. Figure 1 displays the location of the volcano and images during the first days after the eruption. It has to be pointed out that this natural tracer release has provided an exceptional opportunity to perform a detailed monitoring of an anticyclonic eddy. Low and high-resolution satellite images obtained from MODIS, MERIS and WorldView sensors were systematically processed [20] to provide information on the concentration of a number of marine parameters: chlorophyll-a concentration, suspended matter, PIC, POC, *etc*. These oceanographic remote sensing data also played a fundamental role during field campaigns guiding the Spanish Government oceanographic vessel to the appropriate sampling areas [21].

Traditional eddy detection methods using altimeter data can be categorized into three classes [22]: (i) those based on a physical parameter, usually the Okubo–Weiss approach; (ii) those based on flow geometry, with the winding-angle approach being a representative method that identifies eddies by clustering closed or spiral streamlines; and (iii) those based on sea surface height or level anomaly in which a threshold is always required to delimit eddy dimensions. Several thresholds have been applied, typically ranging from 6 cm to 10 cm, depending on the region of study and size of the structures to consider.

In our work, as we deal with higher resolution optical ocean colour data, the detection approaches are different, mainly addressing segmentation techniques. Segmentation is performed based on different attributes of an image such as size, colour, texture *etc*. Several image segmentation techniques have been developed for a variety of applications in various fields of image analysis [23,24,25,26,27], from feature-based approaches, such as clustering and histogram thresholding, as well as spatial domain-based methods, such as region growing-splitting-merging, energy-driven active contours, graph cuts, and watersheds. Our methodology for the automatic and precise detection of oceanographic features is based on an initial structure detection followed by a fine detail growing process.

This paper illustrates the capabilities of multispectral satellite remote sensing systems to improve the understanding and monitoring of submarine volcanic processes and, mainly, it presents a novel image processing methodology for the segmentation of oceanographic structures. To that respect, automated detection methods are essential for understanding the complex movements and the dynamic characteristic of mesoscale eddies.

The organization of contents is structured as follows: Section 2 includes de satellite imagery used and describes the two-steps segmentation algorithm. Section 3 presents the main results. Finally, Section 4 summarizes the main conclusions.

## 2. Methodology

### 2.1. Ocean Colour Imagery

Satellite data from AQUA/TERRA-MODIS and ENVISAT-MERIS sensors have been the main source of remote sensing image information for monitoring El Hierro submarine volcanic processes. The NASA Moderate-resolution Imaging Spectroradiometer (MODIS) has a viewing swath width of 2330 km, it images the Earth in 36 spectral bands, ranging from 0.405 µm to 14.385 µm, and it collects data at three spatial resolutions: 250, 500, and 1000 meters. The ESA Medium Resolution Imaging Spectrometer (MERIS) has a swath width of 1,150 km, measuring the solar radiation reflected by the Earth in 15 spectral bands from 0.4125 µm to 0.9 µm and with a pixel field of view of 0.019°, generating full resolution data at 300 m resolution.

We have implemented a methodology for providing multitemporal and multisensor imagery information, at 1000 m (MODIS) and 300 m (MERIS) resolution, on the concentration of specific oceanographic parameters, mainly chlorophyll-a (*Chl-a*) concentration and diffuse attenuation coefficient (*K_d_*).

The diffuse attenuation coefficient is an important water property related to light penetration in the blue-green wavelengths of the spectrum. It can provide valuable information about the transparency or the amount of dissolved and suspended materials in the water column. Satellite observations of this coefficient, at the wavelength 490 nm, are an effective method to provide turbidity maps for ocean and coastal waters at high spatial and temporal resolutions. Several empirical and semi-analytical models have been developed to estimate *K_d_*; however, they are generally applicable to clear open ocean waters. Recently, improved models have appeared [28] mostly applicable to turbid coastal waters. In our case, satellite estimations of the diffuse attenuation coefficient and chlorophyll-a concentration under these abnormal conditions were assessed [20]. After comparing *in-situ* measurement with *Chl-a* products obtained using the MODIS and MERIS algorithms, we concluded that chlorophyll estimations were erroneous in such areas with extreme turbidity. On the other hand, estimations of *K_d_* using common operational algorithms were not fully accurate due to the physical-chemical alterations provoked by the underwater volcano. For this reason, for MODIS and MERIS, we obtained and validated improved *K_d_* models for coastal turbid waters [20]. As a consequence, only *K_d_* products have been considered appropriate for the monitoring of the oceanographic structures during the event.

**Figure 2:**
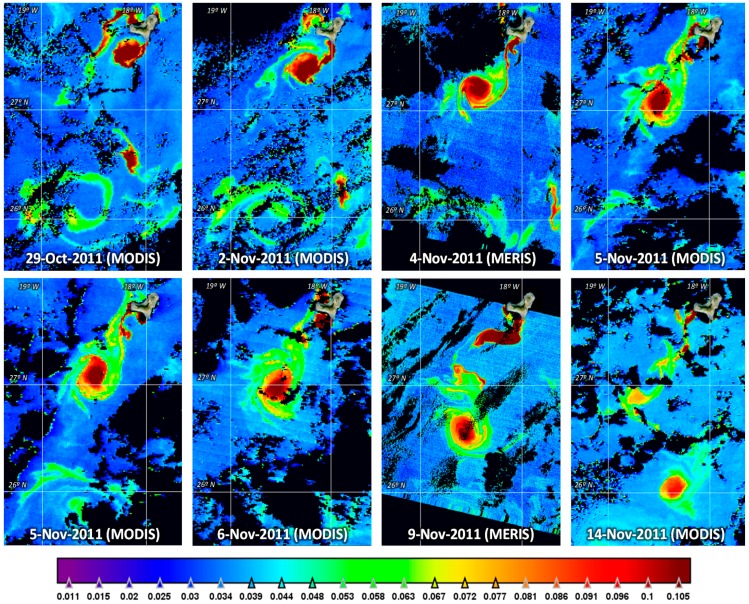
Multitemporal *K_d_* product generated from MODIS (Terra and Aqua) and MERIS (ENVISAT) data during October and November 2011.

Using the mentioned algorithms, we produced periodic *K_d_* maps during the timeframe of the volcanic eruption to allow the monitoring of the plume evolution and other generated structures. As an example, Figure 2 presents multitemporal and multisensor *K_d_* maps of the plume and the eddy progression during specific days of October and November 2011.

Apart from the regular monitoring of the volcano activity with the provision of all cloud free oceanographic products to the governmental and research users, a database composed by *K_d_* MODIS and MERIS images of November 2011, as shown in Figure 2, displaying the evolution of the eddy is used to describe the segmentation methodology developed.

On the other hand, the submarine volcano was also monitored using medium and high resolution data. In particular, the very high resolution Worldview-2 satellite was selected due to its excellent characteristics having eight multispectral bands with 2 m resolution. Thus, WorldView-2 spectral bands were atmospherically corrected and deglinted prior to obtain a very high-resolution *K_d_* model. This novel model was developed and validated using matchup data sets with MERIS, MODIS data, *in-situ* transmittances measurements and a seawater radiative transfer model [20]. This extraordinary resolution allows the analysis of the marine structures at the highest level of detail.

**Figure 3:**
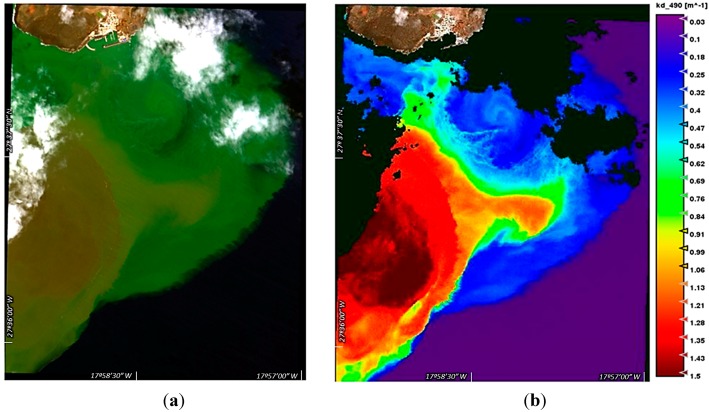
Worldview-2 image of October 26, 2011: (**a**) colour composite (bands 5-3-2) and (**b**) very high resolution diffuse attenuation coefficient map (clouds masked in black).

Figure 3 displays a colour composite WorldView-2 image acquired on 26 October. The image was pre-processed to remove the atmospheric and sun-glint effects in order to reliably obtain the reflectance emitted from the sea surface. We can notice the brown patch in the volcano cone location and greener waters indicating the presence of lower concentrations of volcanic material. Thus, we can see extremely turbid waters with values above 1.5 m^−1^ in the centre of the eruption and gradually decreasing in the surroundings. The diffuse attenuation coefficient was estimated using our improved algorithm [20].

Unfortunately, due to the large horizontal extension of eddies, their continuous monitoring and tracking using this source of information is not suitable and extremely expensive. WorldView-2 was programmed to collect data during these months but, unfortunately, only the displayed image was appropriate (October 2011), while for the rest, the volcano activity had temporarily stopped or was very low in the sensed area.

### 2.2. Methodology for the Precise Segmentation of Eddies

**Figure 4:**
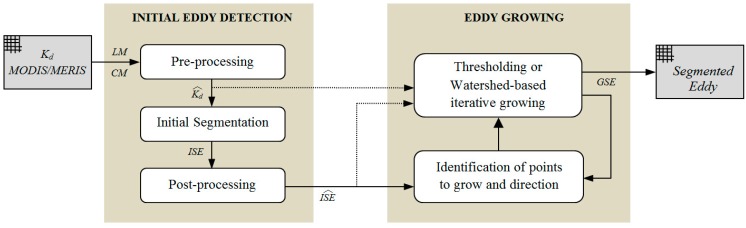
Methodology for the automatic segmentation of eddies using optical imagery.

Methods for detecting eddies are very important and can help to better understand the processes involved in eddy generation, evolution and decaying, as well as their impacts on marine environment and climate change. In addition, such eddy segmentation allows for the evaluation of the numerical modelling performance in terms of eddy centre, positions, sizes and motion. The precise detection approach we have developed is presented in Figure 4. The methodology is composed by two steps:

(i) Initial eddy detection: First, a pre-processing is applied to the MODIS/MERIS *K_d_* images. Next, the initial segmentation stage is applied with the possibility to select different clustering and thresholding algorithms. Finally, a post-processing is required to remove isolated noisy pixels or to fill holes in the eddy structure due to clouds. After the initial detection, the structure is well identified; however the fine detail of the emerging filaments cannot be preserved without incorporating unwanted areas.
(ii) Eddy growth: The precise delimitation is of fundamental importance when analysing the structure and its evolution, so controlled growing techniques are applied only where filaments appear. Points to grow and their directions are found using the skeleton of the initial structure detected. At each point, a window with the corresponding orientation and size masks the image and new pixels are added to the structure after the application of thresholding or watershed region growing algorithms. This process is iteratively applied to achieve the desired fine segmentation.

The different modules that integrate the methodology are next explained in detail.

#### 2.2.1. Initial Eddy Detection

##### Pre-Processing

The proper segmentation of eddies is interfered by the presence of clouds, land, *etc*. So, a cloud mask (*CM*), as well as the land mask (*LM*) are applied to the geometrically corrected (*GC*) *Kd* image. Despite the good performance of the cloud detection algorithm [29], in terms of detection and false alarm probabilities, not all the existing cloud pixels are masked. Therefore they may appear as noise in the segmented image. In order to minimize its effect, an optional 3 × 3 median filtering (*median*) can be applied to the corrected and masked image. This filtering method has also been selected because of its smoothing effect without edge deterioration. Thus, the pre-processed image ($\hat{K_{d}}$) is obtained as follows:

(1) $$\hat{K_{d}}(x,\;y)=median\left[GC\left\{K_{d}\left(x,y\right)\right\}\cap^{\text{​}}CM\left(x,y\right)\cap^{\text{​}}LM(x,y)\right]$$

##### Initial Segmentation

Several segmentation algorithms have been developed over the years, however oceanographic structures are difficult to be properly detected with conventional algorithms. After testing a variety of techniques and analysing their results with expert oceanographers, the following methods for the preliminary eddy segmentation have been selected: *K-means Clustering, Fuzzy K-means Clustering, Mean Shift Clustering and Thresholding*. In [30,31] a detailed evaluation of 36 automatic global and local thresholding techniques was performed and the best methods belonging to different categories (histogram shape-based, clustering-based, entropy-based, object attribute-based, spatial methods, *etc*.) were incorporated to the eddy segmentation tool. In our study, satisfactory results to segment marine structures are achieved by techniques like Riddler, Otsu, Gaussian Mixture Modelling, Pun or Li. In particular, Otsu algorithm provides an intermediate, yet accurate, solution and it can be selected as a compromise technique, providing robustness and excellent results for the majority of images. After the application of the segmentation algorithm, we get a binary image corresponding to the Initial Segmented Eddy (*ISE*):

(2) $$ISE(x,y)=segmentation\left[\hat{K_{d}}\left(x,y\right)\right]$$

**Figure 5:**
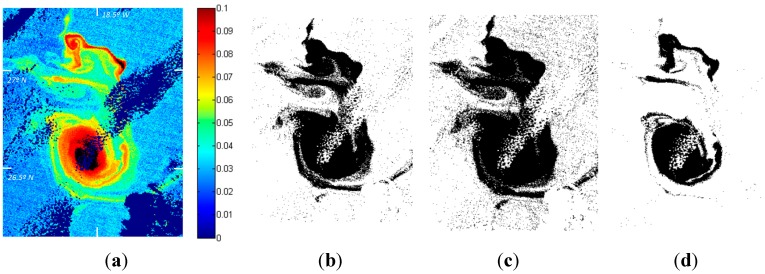
Structures detected by different segmentation algorithms: (**a**) *K_d_* pre-processed imagery; (**b**) K-means; (**c**) Fuzzy K-Means; and (**d**) Otsu.

Some examples of possible automatic segmentations are included in Figure 5. As it can be appreciated, oceanographic structures are not simple to automatically detect due to the fact that gradients are weak, generating several unconnected border lines and making of great difficulty the structure identification based on edge techniques; the presence of noise, mainly due to atmospheric phenomena; the low signal received at sensor level; and the strong morphological variation that impedes an accurate geometric representation and the absence of a valid analytical model for the structures.

**Figure 6:**
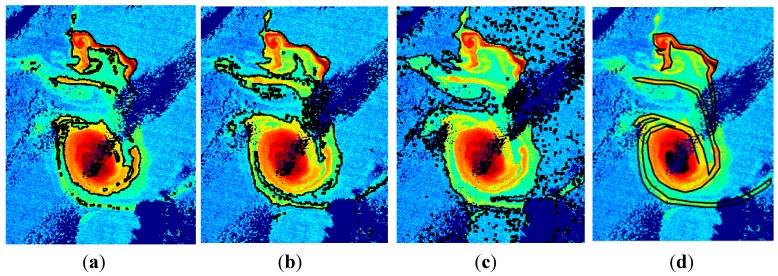
Structures detected by manual thresholding algorithms with global *K_d_* threshold values of: (**a**–**c**) 0.06, 0.05 and 0.04, respectively; (**d**) Segmentation desired by the oceanographers.

Trying a segmentation using manual thresholding techniques provides the results included in Figure 6a–c. It can be appreciated that even using a manual procedure and adjusting the *K_d_* threshold to different values (0.04 to 0.06) it is not possible to achieve at the same time the fine detail of the complete filaments without adding undesired lower levels of *K_d_.*

##### Post-Processing

The segmented image includes the eddy plus additional artefacts due to the presence of noise, isolated turbid water zones and the remaining cloud pixels not properly masked previously. Thus, a post-processing step is required consisting mainly on the application of morphological operators [32]. A closing operator (∅), using a structuring element (typically of size 3 pixels) to smooth and connect pixels, mainly belonging to broken filaments; followed by a removal of holes (*RH*) operation usually to fill cloudy pixels belonging to the eddy. The resulting initial segmented eddy ($\hat{ISE}$) is obtained as follows:

(3) $$\hat{ISE}(x,y)=RH\left[\emptyset \left\{ISE\left(x,y\right)\right\}\right]$$

An additional capability included in the methodology is the elimination of isolated objects whose size is smaller than a threshold defined by the user.

#### 2.2.2. Eddy Growing

After the preceding tasks, the structure is identified and, selecting the corresponding parameters, it is possible to achieve different levels of segmentations to get the desired eddy detection. Unfortunately, none of the tested methods is able to produce the results required by our oceanographers, as presented in Figure 6d, who want to preserve the core of the eddy but also to perfectly delimitate the emerging filaments but without including noise associated to water with less turbidity surrounding its core. The precise delimitation of the filaments provides fundamental information about the submesoscale variability such as the filamentation of the eddy not observed before with such. In addition, such precise delimitation is essential to perform a comparison of a model outcome with observational data from remote sensing imagery.

To achieve this fine segmentation, a two-step procedure has been developed, as shown in Figure 4. It allows the application of controlled growing techniques but only in regions where filaments appear. The procedure is as follows:

##### Identification of Points to Grow and Direction

**Figure 7:**
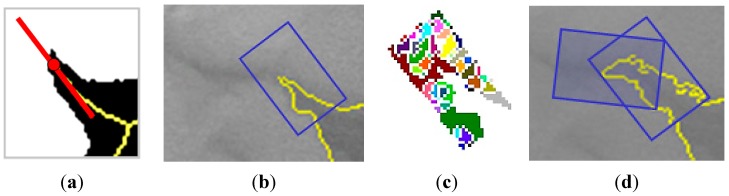
Add Growing process: (**a**) Growing point and direction; (**b**) growing window; (**c**) watershed regions and; (**d**) new structure after the application of watershed and region growing and subsequent growing window.

First, the skeleton (*SK*) of the segmented eddy is used to identify the points to grow and it provides, as well, the growing direction (see Figure 7a in red colour). In this work, the skeleton is obtained by sequential iterations of morphological thinnings to avoid the appearance of spurious branches due to boundary irregularities.

The growing points (*GP*) are obtained by selecting the end-points of the branches of the skeleton [32], as:

(4) $$GP(x,y)=end\_points\left[SK\left\{\hat{ISE}\left(x,y\right)\right\}\right]$$

The growing directions are determined by the angle of the first order polynomial that better fits, in a least-squares sense, the medial axis in the neighbourhood of each growing point.

##### Segmentation-Based Iterative Growing

For each point to grow, a window with the appropriate orientation and size (related with the typical dimensions of the filaments) is generated and used to mask the previous segmented image, as displayed in Figure 7b. Next, to add new filament pixels to the structure within the growing window, different segmentation strategies have been tested. We have incorporated two possibilities [30]:

*Thresholding techniques* use a threshold for each window that is applied to get the grown segmented eddy (*GSE*), as:

(5) $$GSE^{k+1}(x,y)=GSE^{k}(x,y)\cup^{\text{​}}\overset{GP_{max}^{k}}{\underset{i=1}{\sum }}Thr\left[w_{i}(x,y)\cap^{\text{​}}\hat{K_{d}}(x,y)\right]$$

where is the grown structure at the k-iteration (where $GSE^{k}$ is the grown structure at the k-iteration ($GSE^{0}=\hat{ISE}$), *GP^k^* are the growing points obtained from the grown structure at the k-iteration, *w* is the window generated at each growing point and *Thr* represents the thresholded result after applying the corresponding approach. Specifically, two different approaches have been included to decide the appropriate threshold: (i) its value is automatically obtained, for each window, using one of the automatic thresholding methods as previously indicated; or (ii) the threshold is the minimum *K_d_* value allowed to be incorporated to the eddy during the growing process.

*The watershed segmentation* [32], has the advantage that resulting boundaries form closed and connected regions and the union of all the regions forms the entire image, however produces over-segmentation. In our method that is not an inconvenience, as we analyse small windowed regions of the image, and having the maximum number of regions is desirable.

We apply the watershed segmentation to every windowed portion of the image, once the previously detected structure within the window has been excluded. Note that the growing process is guided towards the direction defined in the previous step. Figure 7c shows the regions obtained when applying the watershed transform. Next, only those regions satisfying a spatial proximity criterion to the detected structure will be good candidates to be added, as shown in Figure 7d. The proximity criterion preserves regions falling within the crown generated after subtracting the dilated structure from the original structure. In summary the growing strategy is obtained by:

(6) $$GSE^{k+1}(x,y)=GSE^{k}(x,y)\cup^{\text{​}}\overset{GP_{max}^{k}}{\underset{i=1}{\sum }}RG\left[SP\left\{WS\left(\left[w_{i}\left(x,y\right)\cap^{\text{​}}\hat{K_{d}}\left(x,y\right)\right]\cap^{\text{​}}\left[w_{i}\left(x,y\right)\cap^{\text{​}}GSE^{k}(x,y)\right]\right)\right\}\right]$$

where *RG* indicates the region growing technique, *SP* is the spatial proximity function applied and *WS* represents the watershed transform.

The complete structure growing process previously described at Equations (5) or (6) is iteratively applied until no new pixels are added to the main structure or the required number of iterations has been completed (Figure 7d). 

Finally, it is worth to mention that there are two specific modules, at the output of each step of the methodology, to analyse and extract the maximum information about the eddy (dimensions, area, eccentricity, centre coordinates, *etc*.) and its filaments (length, width, *etc*.), whose results are included in next section.

## 3. Results and Discussion

A regular multidisciplinary monitoring has been carried out by processing all the available MODIS and MERIS data with the goal to get different water quality products in order to quantify environmental impacts caused by the submarine eruption of El Hierro Island.

As indicated, the volcano has provided a unique opportunity to study the marine structures appearing in the Canary Eddy Corridor. To that respect, a novel 2-steps methodology has been developed. The method incorporates different segmentation algorithms and region growing strategies providing enough flexibility to allow the oceanographers to get the best result.

**Figure 8:**
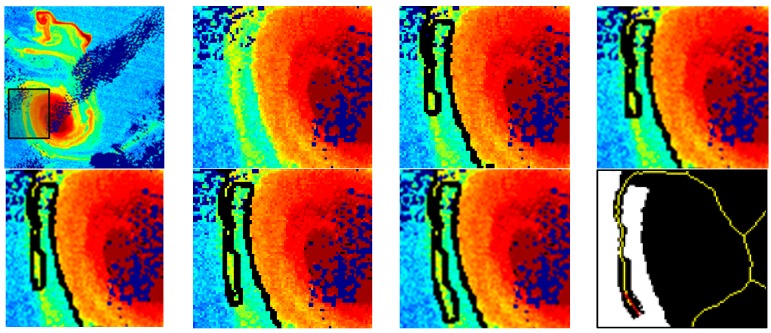
Some results of the iterative growing process included in the Eddy Growing Module for a particular filament. Skeleton and growing direction after the last iteration.

Figure 8 presents the detailed results of the growing process for a portion of the MERIS *K_d_* image applying the automatic threshold-based approach (Otsu algorithm). Only a few iterations are presented to illustrate the growing process on a specific filament. In the last figure, the skeleton (yellow) and the growing direction (red) are included. 

**Figure 9:**
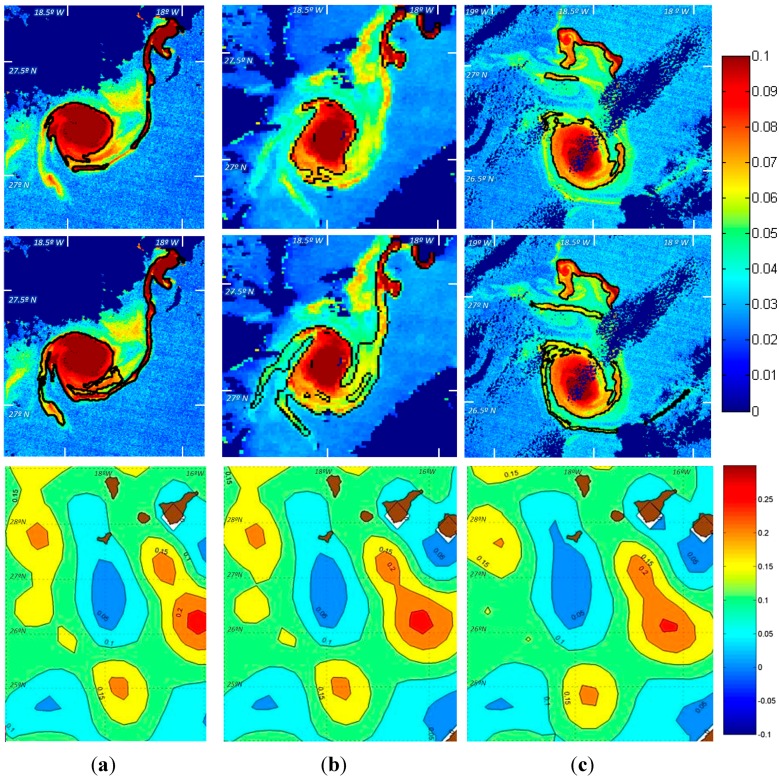
Add Contours of the eddy and its filaments, overlaid on the MERIS and MODIS *K_d_* images in m^−1^, after the initial segmentation (first row) and the structure growing (second row) modules; and Sea Surface Height Anomaly product in cm (last row): (**a**) 4 November 2011; (**b**) 5 November 2011 and (**c**) 9 November 2011.

Figure 9 presents one example of the result achieved for the detection of eddies in three *K_d_* images processed from the MODIS and MERIS sensors. The first row includes the contour of the eddy after the initial detection module while the final contour after the growing stage is shown in the second row. We can appreciate that filaments have been properly captured, even those having low diffuse attenuation coefficient values (greenish tones), without the inclusion of noise associated to other areas having similar low *K_d_* levels (tonalities). Notice that the spiraling filaments detach form each vertex of the elliptical shaped core. Image sequences also show how the spiraling like eddy rotates around its centre of mass. Therefore, superposed to water parcel orbits around the eddy centre, there is a low frequency anticyclonic rotation of the ensemble constituted by the elliptical core and attached filaments. This low frequency rotation may be appreciated by the deformation of the filaments through sequences of Figure 9. In Figure 9 we have also included the sea surface height anomaly fields obtained from altimeters [33] for the same days, just to demonstrate the impossibility of undertaking submesoscale analysis using this sort of coarse data.

Detailed information about the morphology and location of the core of the eddy has also been extracted for the images under analysis. Table 1 includes the corresponding parameters and their specific value for each date. Apart from this data extracted after the first step of the methodology, it is also possible to measure the length and width of each filament after the growing process has been completed. Theoretical studies for an isolated elliptical vortex predicts that when semi-major axis, semi-minor axis aspect ratio is above the threshold value of 3 the filamentation process starts. Table 1 values indicate that aspect ratio do not exceed 1.7 suggesting that the filamentation is not related with it elliptical shape instability. Instead it may be linked to the entrainment of the El Hierro boundary layer during its generation stage, as showed by numerical models on flow perturbation by an Island [34]. Theoretical studies also predict that the process of filamentation is accompanied by an axisymmetrization of the vortex [35]. The circulation transported into the filaments, although a small fraction of the total breaks the symmetry and is the chief cause of axisymmetrization. To our knowledge, this process has not been yet identified in the nature. Eccentricity values of Table 1 clearly indicate that the eddy evolves toward a circular shape denoting that the process of axisymmetrization is taking place. It is worth mention that the methodology implemented has also been tested with the remaining available MODIS and MERIS imagery providing an excellent performance and allowing the reliable segmentation of the existing eddies and the extraction of the required information.

**Table 1:** Extracted Parameters Describing the Eddy at Different Dates.

Date	Centroid		Area (km^2^)	Perimeter (km)	Semi-Major axis (km)	Semi-Minor axis (km)	Ecc.
	Latitude	Longitude					
November 4	27.1819°	−18.4426°	623	187	15.67	13.23	0.54
November 5	27.0866°	−18.5024°	680	152	18.67	12.61	0.62
November 9	26.5727°	−18.5751°	697	174	17.22	13.14	0.65

## 4. Conclusions

In October 2011, an underwater eruption gave rise to a novel submarine volcano south of the island of El Hierro, in the Canary Islands, Spain. During the eruption, large quantities of mantle-derived gases, solutes and heat were released into the surrounding waters. Through the months the eruption lasted, satellite remote sensing imagery has been of fundamental importance to guide the governmental oceanographic vessel during field campaigns and also to monitor the impact of the eruption on the marine ecosystem.

Specifically, the periodic processing of MODIS and MERIS data was performed to provide information on the concentration of a number of biological, physical and chemical parameters. Apart from low-resolution data, the volcano activity and effects were also examined using very high resolution from the Worldview-2 satellite.

On the other hand, the eruption of the underwater volcano has provided an exceptional source of tracer that allowed the study a variety of structures. In particular, eddies are of great interest as they can exert an important role on the global climate and local oceanic dynamic due its influence in the transport of ocean heat and momentum, as well as, in the enhancement of vertical shear mixing. It is important to emphasize that the Canary Islands’ area is a very active zone of long-lived eddies.

Thanks to the volcanic tracer, detailed studies could be undertaken using ocean colour imagery. In our work we have considered MODIS and MERIS images and, in particular, diffuse attenuation coefficient products have been processed to allow the analysis of such structures at much higher resolution. The material released by the volcano has provided a unique high resolution picture of an anticyclonic mesoscale eddy. This has allowed observing for the first time in the nature the process of filamentation and axisymmetrization predicted by theoretical studies and numerical modelling.

To that respect, a novel 2-step methodology has been developed. It is composed by an initial structure detection module followed by a fine detail growing process. The methodology obtains the initial eddy segmentation and achieves the maximum detail of the structure, especially for the emerging filaments, after the iterative identification of the points to grow and the subsequent application of growing strategies. The method incorporates different segmentation algorithms and region growing techniques providing enough flexibility to allow the oceanographers to get the best result and to obtain detailed information about the morphology and location of the core of the eddy, as well as the length and width of each filament.

This detection approach has been validated over a database of MERIS and MODIS oceanographic products and it has demonstrated an excellent performance and robustness to noise and weak gradients. Specifically, it has proven to properly capture the filaments, even those having low *K_d_* values, without the inclusion of noise associated to other areas having similar low *K_d_* levels.

In summary, the underwater volcano has allowed the precise analysis of an anticyclonic eddy, providing valuable information at a resolution that cannot be reached using sea level anomaly fields from altimeters.
